# Cellulose-Based Electrochemical Sensors

**DOI:** 10.3390/s25030645

**Published:** 2025-01-22

**Authors:** Muhammad Sheraz, Xiao-Feng Sun, Adeena Siddiqui, Yongke Wang, Sihai Hu, Ran Sun

**Affiliations:** School of Chemistry and Chemical Engineering, Northwestern Polytechnical University, Xi’an 710129, China; sherazmuhammad@mail.nwpu.edu.cn (M.S.); aadeenasiddiqui@gmail.com (A.S.); shidaijiuji@mail.nwpu.edu.cn (Y.W.); husihai@nwpu.edu.cn (S.H.);

**Keywords:** electrochemical sensors, biocompatibility, cellulose-derived materials, environmental monitoring, medical diagnostics

## Abstract

Among the most promising areas of research, cellulose-based electrochemical sensors stand out for their intrinsic properties such as abundance, biocompatibility, and versatility. This review is concerned with the integration and application of cellulose-derived materials in electrochemical sensors, pointing out improvements in sensitivity, selectivity, stability, and functionality for a wide variety of applications. The most relevant developments on cellulose-based sensors have been concentrated on nanocellulose composite synthesis, advanced cellulose modification, and the successful embedding in wearable technologies, medical diagnostics, and environmental monitoring. Considering these, it is worth mentioning that significant challenges still need to be overcome regarding the scalability of production, selectivity improvement, and long-term stability under real operational conditions. Future research efforts will concern the union of cellulose-based sensors with the Internet of Things (IoT) and artificial intelligence (AI) toward wiser and more sustainable health and environmental solutions. Correspondingly, this work puts cellulose in the front line among the most perspective materials for enabling the development of eco-friendly and high-performance sensing technologies.

## 1. Introduction

**Figure 1:**
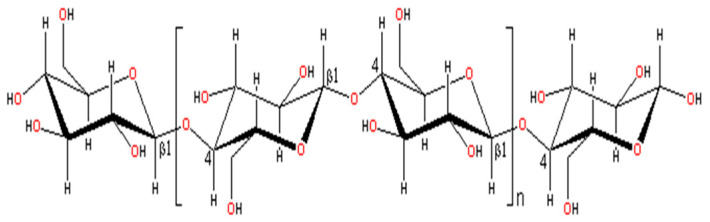
Chemical structure of cellulose.

Polysaccharides such as cellulose, chitosan, alginate, and hyaluronic acid encompass enormous biomaterials. This is because of their inherent abundance on Earth and impressive physicochemical and biological attributes [1,2]. As the most abundant biopolymer on Earth, cellulose and its derivatives have been among the most studied materials in several biomedical applications, including hydrogels, aerogels, films, and fillers, due to their outstanding properties and biocompatibility [3,4,5,6,7,8]. Cellulose is a linear homopolysaccharide consisting of β-1,4-linked glucans and has abundant hydroxyl groups on its surface which have a formula of (C_6_H_10_O_5_)*_n_*. The chemical structure of cellulose is shown in Figure 1. It possesses a high degree of symmetry within its amphiphilic molecular structure [9,10,11]. Its high surface area and excellent biocompatibility, with tunable functional groups, make it a prime candidate for improving sensor performance. Its renewability, together with features tailored for a range of sensor designs, positions cellulose as an ecologically friendly yet highly efficient solution for the development of state-of-the-art, eco-friendly sensing technologies. Chemical modifications include carboxylation, esterification, and grafting of cellulose, which enhances its ability to increase electron transfer, sensitivity, and specificity in the selective identification of target analytes. By modifying cellulose, it becomes highly adaptable material for sensors, capable of detecting environmental pollutants and biomolecules with improved efficiency [12,13,14]. The source of cellulose will affect its structure and properties, and these sources are as varied as plants [15] including cotton, sisal, and wood, algae [16] such as valonia, and bacterial sources including *Komagataeibacter* sp. [17]. There have been various studies that report on the extraction of cellulose from numerous biomass sources, pointing to various methods and approach developments in past years [11,18,19,20,21,22,23,24,25,26].

Generally, the method for electrochemical sensing involves monitoring analyte–receptor interactions within an electrolyte solution using either potentiometry or amperometry. However, fouling—that is, the accumulation of large organic surfactants, proteins, or reactions on the electrode surface—and poor selectivity are major drawbacks for these sensors. As a result, many of them are not highly effective in some clinical and environmental applications. On the other hand, cellulose is considered one of the most promising membrane materials in overcoming such problems since it has very minimal fouling and better selectivity [27,28,29,30]. Recently, the combination of cellulose with carbon-based nanomaterials such as graphene [31], graphene oxide (GO) [32], single-walled carbon nanotubes (SWCNTs) [33], multi-walled carbon nanotubes (MWCNTs) [34], and nanodiamonds [35] has also been a very promising approach for the development of nanocomposites for various electrochemical applications, including amperometric sensing. Transparent, ultra-strong, and printable conductive nanocomposites have been developed by incorporating 2,2,6,6-tetramethylpiperidine-1-oxyl (TEMPO)-oxidized cellulose nanofibrils (TOCNFs) with SWCNTs [33]. Recently, the stability of four different nanocomposites prepared using fibrillar nanocelluloses with different functional groups (sulfate, carboxylate, and amino-silane) and MWCNTs was tested. These nanocomposites exhibited very good stability in a wide potential window (−0.6 to +1 V) in various electrolytes [34].

In recent decades, all kinds of smart electronic devices have been developed and extended to various applications, including smart human motion tracking, flexible human–computer interfaces, touchscreen displays, energy generation, storage system, smart glazing, or actuators [36,37,38,39,40,41,42,43]. All involved sensors have been located as one of the most significant directions in this area and continue to attract broad scientific concern because of their extraordinary ability to respond to the effects of physical forces, chemical substances, and biological analytes. These sensor elements transform this input energy into an accessible, recognizable, and manageable form of a signal [44]. Based on the remarkable electrochemical properties of cellulose, we will discuss cellulose-based electrochemical sensors, focusing on their development, design, and applications.

## 2. Cellulose-Based Materials for Electrochemical Sensors

### 2.1. Types of Cellulose Used in Electrochemical Sensors

Naturally, cellulose fibers contain regions of crystalline and amorphous nature. Microcrystalline cellulose (MCC) is a refined, partially depolymerized cellulose obtained from α-cellulose source [45]. However, MCC can be produced using several techniques which include reactive extrusion [46], enzyme-mediated [47], and acid hydrolysis [48,49,50,51] techniques; all these techniques remove amorphous regions, retaining the crystalline domains with a more ordered structure. The hydroxyl groups located on the surface of the cellulose and the very well-organized structure of molecules in MCC can interact well with a range of materials, including conducting polymers [52]. MCC is mainly produced from wood and cotton [53], and its application also extends to the pharmaceutical [54], medical [55], food [56,57,58], and cosmetic industries [58,59] due to its wide range of applications. The usage of MCC as a reinforcement in composites, however, has been less common compared to the usage rate of lignocellulosic fibers; however, in recent years, there has been growing interest in incorporating MCC into composite materials [60,61,62]. Therefore, MCC has become one of the favorite candidates for composite applications because of its renewability, biodegradability, high surface area, low density, and other favorable properties [63]. A surfactant-directed microcrystalline cellulose/polyaniline composite was developed, exhibiting enhanced electrochemical properties [52].

Nanocellulose is highly attractive due to its unique properties, diversified precursors, wide application fields, and biodegradable nature [64]. Based on size, morphology, and synthesis method, nanocellulose can be categorized as cellulose nanocrystals (CNCs) and cellulose nanofibers (CNFs) [65,66,67]. Traditionally, CNCs are produced through chemical routes where hydrolysis produces short rod-shaped particles with diameters between 3 and 10 nm [68,69,70]. Most CNFs have been produced mechanically by ball milling, grinding, and homogenization techniques that generate flexible, fibril-shaped particles [71,72]. Dimensions of both CNCs and CNFs can be reduced using ultrasonication during processing [73,74,75]. In general, CNCs have dimensions smaller than CNFs, which gives them a higher crystallinity index, and hence they can act more effectively as reinforcing agents in various polymer matrices. On the other hand, CNFs represent long flexible fibers with a larger diameter, and due to longer and smaller diameter fibrils, they offer a better reinforcing effect [76,77]. Nanocellulose is invariably combined with active materials to develop its application areas. The synergistic relation between nanocellulose and the involved materials has been key in accomplishing state-of-the-art physical sensors, chemical sensors, or biosensors with high performance in different dimensions. Various associated fabrication techniques enabled the development and construction of nanocellulose-based sensors for a series of applications [78,79,80,81,82,83]. Several reviews have documented the use of nanocellulose in sensor applications [84,85,86,87,88].

Because of the high purity and peculiar physicochemical properties, microbial cellulose synthesized by bacteria known as bacterial cellulose (BC) can be regarded as a source of pure cellulose [89]. Its uses include applications in food [90], biomedical [91,92,93,94], production of bio-based polymers, and nanocomposites [95,96,97]. Only a few species of bacteria are known to synthesize cellulose, and among the prominent ones is *Gluconacetobacter xylinus*, previously called *Acetobacter xylinum* [98]. This Gram-negative, rod-shaped, aerobic bacterium produces cellulose at very high yields like microfibrils, making it a model organism to produce bacterial cellulose (BC) [99]. Other bacterial genera that can produce BC include *Agrobacterium* spp., *Acetobacter* spp., *Azotobacter*, *Rhizobium* spp., *Sarcina*, *Alcaligenes*, and *Pseudomonas* [89]. *Acetobacter*, a genus of vinegar bacteria, is a non-photosynthetic advanced purple bacterium capable of converting glucose, glycerol, sugars, and other organic compounds into pure cellulose [100]. Several different composites of bacterial cellulose have been developed by modifying the bacterial growth conditions, introducing a secondary phase, and including materials like starch, collagen, hydroxyapatite, carbon nanotubes, and graphene [101,102,103,104,105,106]. These additions have enabled an improvement in some properties of high interest, including biocompatibility [102] and electrical conductivity [101,105,107]. Various enzymes, including laccase and heme proteins such as glucose oxidase (GOx) and horseradish peroxidase, have been immobilized into BC-based networks to develop electrochemical biosensors aimed at detecting compounds like H_2_O_2_, hydroquinone, dopamine, and glucose [108,109,110,111,112]. Biosensors made from BC are produced by combining it with various materials, and they are classified according to their origin and potential uses [107,113,114,115]. For example, an electrochemical biosensor was successfully developed using a BC substrate for lactate detection in artificial sweat [28].

### 2.2. Modifications of Cellulose

**Figure 2:**
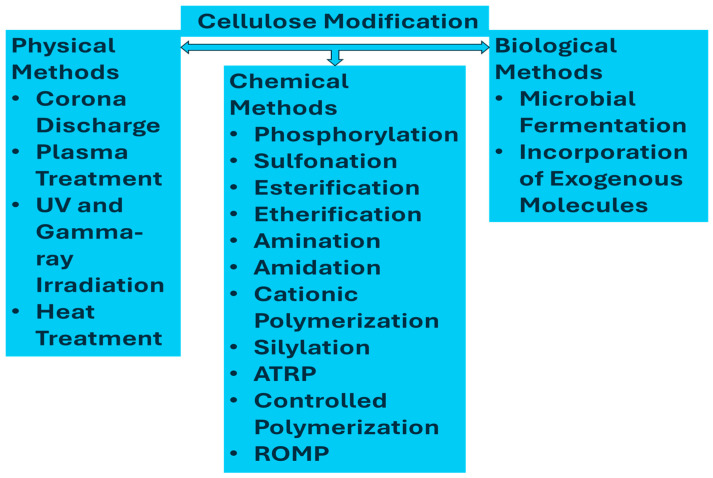
Classification of cellulose modification methods.

The modification of cellulose primarily focuses on improving its adsorption properties by altering its surface. The structure of cellulose consists of tetrahydropyran units connected by 1–4 glycosidic bonds, each containing a hydroxyl (-OH) and primary alcohol (-CH_2_OH) group. During modification, the active hydroxyl group, particularly the one attached to the primary alcohol, is replaced with other functional groups, thereby enhancing the desired properties of the material [116,117]. Classification of the cellulose modification methods is presented in Figure 2. Among the physical methods, corona discharge [118], plasma treatment [119,120,121], UV and gamma-ray irradiations [122,123,124], and heat treatment [125,126] modify the surface properties of cellulose fibers without the involvement of harsh chemicals. Corona discharge treatment includes the introduction of high-energy electrons via high-frequency discharges to facilitate the creation of cavities and micropores on the fiber surface. Oxygen plasma treatment effectively modifies bacterial cellulose membranes by enhancing hydrophilicity and introducing structural changes, while water permeability is reduced, resulting in the improved selectivity of membranes. Gamma radiation or other forms of radiation-based techniques exploit crosslinking within the polymer chain and are useful in altering the properties of cellulose-reinforced biopolymer composites. This results basically in the modification of the composite structure and mechanical behavior of either improved interfacial bonding or a modification of crystallinity. Heat treatment is a process where wood/fiber is exposed to elevated temperatures (150–280 °C) under controlled atmospheric conditions to improve properties such as reduced shrinkage, swelling, moisture content, enhanced decay resistance, and weather durability. However, it also leads to decreased mechanical properties, such as strength and stiffness. Biological methods could be cellulose modification either by microbial fermentation or by the incorporation of exogenous molecules, therefore offering a very effective chemical-free method for bacterial and plant cellulose fibers. These are scalable and quite effective methods [127,128]. With high efficiency and environmental friendliness, the biological process intends to establish bacterial cellulose (BC) functionalization methods through microbial fermentation. It uses glucose that is treated with 6-carboxyfluorescein (6CF-Glc) as a substrate. On this basis, it proposes alternative technologies for modifying bacterial cellulose as a substitute for chemical and physical agents.

Cellulose can also be modified chemically by methods such as phosphorylation [129], sulfonation [130], esterification [131], and etherification [132] to impart better properties. Other methods include amination [133], amidation [134], cationic polymerization [135], silylation [136], atom transfer radical polymerization (ATRP) [137], controlled polymerization [138], and ring opening metathesis polymerization (ROMP) [139]. The phosphorylation of microcrystalline cellulose (MCC) was achieved using phosphoric acid in the presence of molten urea, enhancing the material’s adsorption properties by incorporating phosphate groups that increased reactivity and surface charge. Sulfonation was carried out by treating oxidized cellulose fibers with sodium bisulfite to convert some hydroxyl groups into sulfonic acid groups, which improved the ionic conductivity and selective transport property of the cellulose electrospun fibers. Cellulose nanofibers were esterified with toluene, sulfuric acid as a catalyst, and para-amino-benzoic acid under microwave irradiation and then passed through suction filter, washed with ethanol, and dried. In a one-pot process, cellulose was etherified via a hydroxyl–yne click reaction, forming ether bonds that enhanced UV shielding, mechanical strength, and solvent resistance.

Cellulose was chemically modified by introducing amine groups through reactions with ethylenediamine to increase its total reactivity and network-forming ability in adhesives (known as aminated cellulose or aminated nanocellulose). Amidation was carried out by mixing hydroxy carboxymethyl cellulose (HCMC) with amines at 140 °C with rotary stirring for 20 h, followed by filtration, washing with a solvent, and drying to yield the amidated derivatives. In cationic polymerization, a cationic monomer such as diallyldimethylammonium chloride (DADMAC) was grafted in situ to cellulose fibers using a free radical initiator, increasing charge density and filler retention. Silylation was performed by treating CNCs with hexadecyltrimethoxysilane and perfluorooctyltriethoxysilane to improve hydrophobicity by replacing hydroxyl groups with lengthy chains of alkyl and fluoroalkyl, respectively. Using ATRP, thermo-responsive and fluorescent polymers can be grafted onto cellulose nanocrystals in a metal-free, light-mediated way exploiting 10-phenyl-phenothiazine (PTH) as a photocatalyst. Controlled polymerization allows for the determination of polymer molecular weight, graft length, and architecture so that the polymers may be accurately grafted to the cellulose during chemical modification. Grubbs’ catalyst was used in ring-opening metathesis polymerization (ROMP) to graft polynorbornene onto cellulose fibers to increase hydrophobicity and change structural properties. These modifications increase the reactivity, conductivity, solubility, flexibility, and compatibility of cellulose, hence its applications in many electrochemical sensors.

## 3. Fabrication of Cellulose-Based Electrochemical Sensors

The technology behind sensing platforms has been developed in recent decades, resulting in the creation of robust systems for quick, simple, and cost-effective analysis [140]. This progress has paved the way for the rise of point-of-care testing, which focuses on quantifying various targets in body fluids for diagnostics and health monitoring [141,142,143]. Wearable sensors have emerged as an innovative approach for non-invasive biosensing, particularly for continuous health monitoring, even during exercise [144,145,146,147,148]. Different types of approaches have been employed to enhance cellulose-based electrochemical sensors. Techniques originally introduced for paper-based sensors and nanocomposite fabrication have been applied to improve sensitivity. This has enabled the development of cellulose materials tailored for electrochemical detection.

### 3.1. Paper-Based Electrochemical Sensors

**Figure 3:**
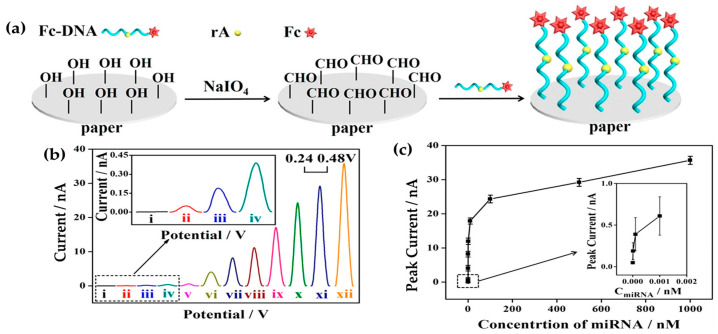
Fabrication of a paper-based electrochemical sensing platform for miRNA: the immobilization of ferrocene-labeled DNA (Fc-DNA) on paper. (**a**) The detection of the lung cancer-specific biomarker miRNA-21 at different concentrations using differential pulse voltammetry. (**b**) DPV currents of the paper-based electrochemical sensor (PES) response to increasing miR-21 concentrations: (i–xii) 0, 10^−15^, 10^−14^, 10^−13^, 10^−12^, 10^−11^, 10^−10^, 10^−9^, 10^−8^, 10^−7^, 0.5 × 10^−7^, and 10^−6^ M, respectively. (**c**) The corresponding calibration curve, with peak current plotted against miR-21 concentration. An inset shows the curve for lower miR-21 concentrations in a dotted square frame [149]. Copyrights: images have been taken with permission from © 2019, American Chemical Society.

Carbon nanotubes and ferrocene-labeled DNA (Fc-DNA) were used to modify paper-based screen-printed electrodes for miRNA detection in serum samples (Figure 3) [149]. DNA immobilization on paper-modified electrodes enhances the reliability and responsiveness of bioassays, hence showing promise for point-of-care (POC) diagnostics. More recently, a composite paper has been fabricated via the traditional papermaking method using cellulose fibers (CFs) for conductivity and GO for electrocatalytic properties [150]. This approach can be efficiently scaled up to create affordable, high-quality, paper-based electrochemical sensors. The composite paper exhibited excellent stability and reproducibility in electrochemical properties with noticeable electrocatalytic activity [27].

### 3.2. Nanocellulose-Based Nanocomposite Electrochemical Sensors

**Figure 4:**
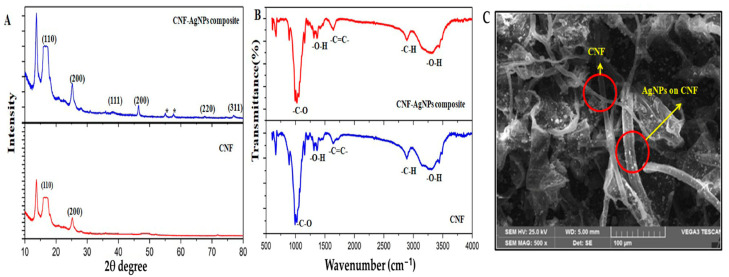
(**A**) XRD pattern showing the structure of CNF and the CNF-AgNPs composite. (**B**) FTIR spectrum comparing the CNF and CNF-AgNPs composite. (**C**) SEM image displaying the morphology of AgNPs and CNF [151]. Copyrights: images have been taken with permission from © 2021, Elsevier.

Nanocellulose-based sensors were created by using cellulose nanofibers from sugarcane bagasse and silver nanoparticles (AgNPs). To create AgNPs, 1 mM silver nitrate was added to the cellulose nanofibers (CNFs). Ultrasonication was then applied, which helped the silver nanoparticles attach to the surface of the CNFs. CNF-AgNPs composite was then applied to modify a graphite electrode. The electrode was polished to a mirror finish, sonicated to remove any particles that were not tightly attached, and coated with a 5 µL solution of the composite before being dried. The finished graphite/CNF-AgNPs sensor was stored at 4 °C and characterized using electrochemical techniques, including electrochemical impedance spectroscopy (EIS). This method effectively produced a nanocellulose-based composite electrode, which showed great potential for electrochemical sensing applications [151]. Figure 4 presents the structural analysis of the CNF-AgNPs composite.

**Figure 5:**
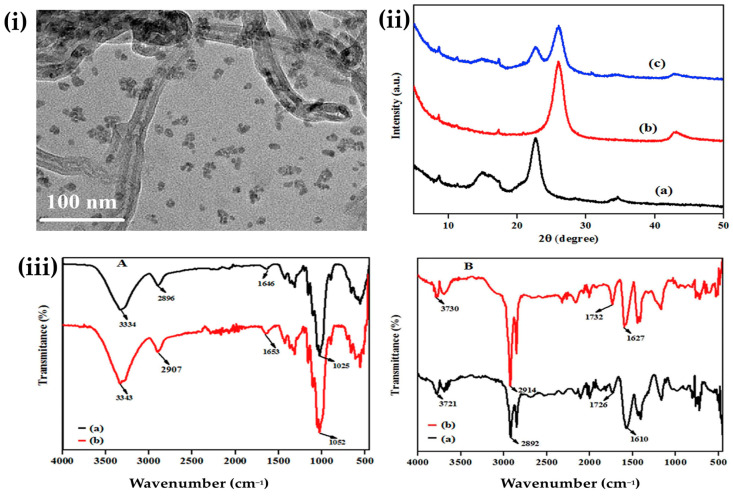
(**i**) TEM image of the nanocellulose/f-MWCNTs composite. (**ii**) X-ray diffraction patterns of (a) nanocellulose, (b) f-MWCNTs, and (c) nanocellulose/f-MWCNTs composite. (**iii**) FTIR spectra of (A) cellulose and nanocellulose and (B) pristine and f-MWCNTs [152]. Copyrights: images have been taken with permission from © 2019, Elsevier.

Shalauddin et al. developed a composite electrode using functionalized multi-walled carbon nanotubes (f-MWCNTs) and nanocellulose (NC) for the electrochemical detection of diclofenac sodium (DCF). F-MWCNTs were prepared by refluxing pristine MWCNTs in a mixture of nitric acid and sulfuric acid in a ratio of 1:3 for 24 h followed by washing and drying. The nanocellulose was prepared from cotton linter using acid hydrolysis with hydrochloric acid and sulfuric acid, and then it was centrifuged, washed, and dried. To modify the electrode, a glassy carbon electrode (GCE) was first polished and then sequentially coated with suspensions of NC and f-MWCNTs. The 5 mM NC solution was applied to the GCE and allowed to dry, and after then, 6 mM f-MWCNTs solution was dropped-cast onto it. The composite electrode was characterized using TEM, FTIR, and XRD to confirm the uniformity and surface structure. The final f-MWCNT/NC/GC electrode demonstrated enhanced electrical conductivity and a larger surface area, which significantly improved its ability to electrochemically detect DCF. To examine the electrochemical properties, cyclic voltammetry (CV), differential pulse voltammetry (DPV), and electrochemical impedance spectroscopy (EIS) were utilized [152]. Figure 5 illustrates the TEM image of NC/f-MWCNTs composite, XRD patterns of NC, f-MWCNTs, and NC/f-MWCNTs composites, and FTIR spectra of cellulose and nanocellulose, as well as pristine and f-MWCNTs.

**Figure 6:**
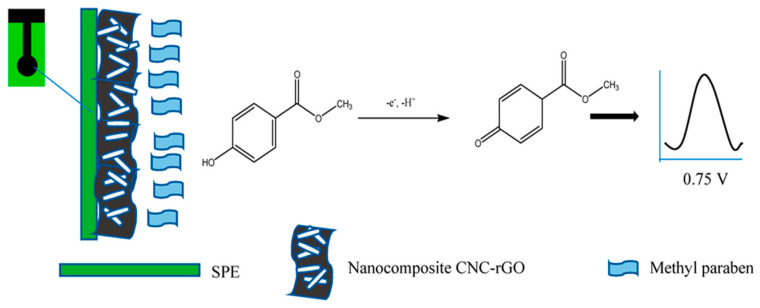
A schematic representation of the sensor’s fabrication process and its detection of MP [153]. Copyrights: images have been taken and reproduced from © 2019, MDPI.

Cellulose nanocrystal–graphene electrochemical sensors were created by modifying electrodes with graphene or their derivatives, like reduced graphene oxide (rGO), and combining them with nanomaterials such as cellulose nanocrystals (CNC). The fabrication process starts by dispersing 1 mg of rGO in ethanol and 1 mg of CNC in deionized water, which are then mixed ultrasonically to form a stable nanocomposite. The composite is drop-coated onto the surface of a screen-printed electrode (SPE) and allowed to air dry at room temperature. To examine the electrochemical properties of both the bare and modified electrodes, methods such as cyclic voltammetry (CV), differential pulse voltammetry (DPV), and electrochemical impedance spectroscopy (EIS) are utilized. The scan rate, pH, and CNC:rGO composite ratios (ranging from 0.5:1 to 3:1) are adjusted to optimize the sensitivity for detecting analytes such as methyl paraben (MP). The sensor’s performance is tested for reproducibility by measuring the current response of five different electrodes and for repeatability by continuously measuring a single electrode’s response. Stability is checked by monitoring the current over a period of 28 days. The potential interference from substances like ascorbic acid, benzoic acid, salicylic acid, and citric acid is tested in mixed solutions. Lastly, the sensor is applied in real-world sample analysis, such as detecting MP in aloe vera cream using the standard addition method, which demonstrates the practical use of the fabricated graphene-based electrochemical sensor [153]. Figure 6 shows a schematic representation of the sensor’s fabrication process and its detection of MP.

## 4. Electrochemical Sensors

### 4.1. Medical and Healthcare Sensors

Microbial nanocellulose (MNC) and screen-printed carbon electrodes (SPCEs) are used to create a wearable sensor platform that can detect biomarkers and harmful metals in sweat in real time [154]. A peptide nucleic acid (PNA)-based electrochemical biosensor for the detection of *Mycobacterium tuberculosis* was designed on a screen-printed carbon electrode modified with NH_2_-rGO/TEMPO-NCC nanohybrid films [155], and a selective cholesterol sensor was also developed using silylated graphene oxide-grafted chemically modified nanocellulose (Si-GO-g-CMNC) with zinc oxide (ZnO) to enhance conductivity [156]. For the determination of phenol, a biosensor was developed using the nanocrystalline cellulose (NCC)/CdS quantum dots (QDs) nanocomposite, which was modified with cetyltriammonium bromide (CTAB) and 3-mercaptopropionic acid (3-MPA) for the immobilization of tyrosinase enzyme (Tyr). Its linear response to phenol was in the range from 5 to 40 µM, while sensitivity and limit of detection (LOD) were estimated as 0.078 µA/µM and 0.082 µM, respectively [157].

A low-cost, reproducible glucose sensor using TEMPO-oxidized cellulose nanocrystals (CNCs) as a matrix was developed. The sensor showed a linear range of 0.1–2 mM, sensitivity of 5.7 ± 0.3 µA cm^−2^·mM^−1^, and a detection limit of 0.004 mM. It maintained 92.3% of its initial response after 30 measurements and had a 1-month shelf life. The sensor was successfully used to monitor glucose consumption in fibroblast cell cultures [158]. A graphene paper functionalized with fractal platinum nanocauliflower was developed for the electrochemical biosensing of glucose and *E. coli* O157:H7. The sensor showed high sensitivity and detection limits for glucose and *E. coli*. It offered fast response times (6 s for glucose, 12 min for *E. coli*) and demonstrated potential for point-of-care biosensing applications [159]. A polypyrrole–cellulose nanocrystal (PPy-CNC) composite with glucose oxidase (GOx) was also developed for glucose sensing. The sensor showed high sensitivity and detection limit, and a range of 1.0–20 mM glucose. It excluded interfering substances and maintained over 95% of its initial response after 17 days, with good reproducibility and stability [160].

Interestingly, an ultrasensitive sandwich-type electrochemical immunosensor was constructed using graphene–perylene-3,4,9,10–tetracarboxylic acid–nanocellulose–Au nanocomposites to establish the detection platform of avian leukosis virus subgroup J (ALV-J), which displayed wide detectable ranges with low limits of detection. The prepared sensor was characterized by high sensitivity, selectivity, reproducibility, and stability, having good application prospects toward clinical ALV-J detection [161].

### 4.2. Environmental Sensors

The development of a novel electrochemical sensor was presented for detecting Hg^2+^ in water, utilizing a hybrid nanocomposite of reduced graphene oxide (rGO), cellulose nanowhiskers (CNWs), and polyamide 6 (PA6) nanofibers. This sensor demonstrated enhanced charge transfer, high sensitivity, excellent stability, selectivity, a low detection limit, and a wide dynamic range for mercury detection [162]. Bacterial cellulose–gold nanoparticle (BC-AuNP) hybrid nanofibers were synthesized and used to create an electrochemical biosensor for hydroquinone detection. The sensor exhibited excellent electrocatalysis for dopamine, with a low detection limit of 5.71 nM and a wide linear range (30–100 nM). It showed good repeatability, selectivity, and stability and was successfully applied to detect hydroquinone in lake water samples [109]. The electrochemical behavior of atrazine (ATZ) was investigated in a Britton–Robinson buffer at pH 4.3 using a cellulose-modified pencil graphite electrode. Characterization of the sensor was carried out by electrochemical impedance spectroscopy (EIS) and cyclic voltammetry (CV), presenting a reduction peak at 880 mV in CV. A linear response of ATZ was obtained in the range from 5.0 ng/mL to 320 ng/mL, with a detection limit of 0.008 ng/mL. The sensor was of high selectivity, sensitivity, and good reproducibility. It has been successfully applied in the detection of ATZ in drinking water, giving recoveries of 98.8–99.1% [163]. An electrochemical biosensor based on nanocellulose was developed to selectively and sensitively detect xanthine (XA) for fish spoilage monitoring. The nanocellulose, prepared from raw cotton, had xanthine oxidase immobilized onto it. This biosensor showed a linear range of detection between 3 and 50 µM, while the limit of detection was 47.96 nM, and it was highly sensitive. Stability, after one month, was excellent, up to 97%, as well as high selectivity, which resulted in good agreement with commercial methods utilizing spectrophotometry [164]. Films of cellulose nanocrystals–polyaniline were developed as highly sensitive electrochemical sensors, responding to various kinds of stimuli. The incorporation of polyaniline (PANI) into the chiral nematic CNC films allowed for changes in color that depended on various environmental factors such as humidity, pH, and organic solvents. The incorporation of glucose enhanced the flexibility of these films without breaking the chiral structure. These films demonstrated distinct color and conductivity changes depending on the conditions and, therefore, were good candidates for use in multi-sensing applications. A two-layer CNC-PANI film was also prepared for use as a conductive wire that completes a circuit upon stimulation and can find practical applications in environmental monitoring or reaction condition warnings [165]. Some examples of cellulose-based electrochemical sensors are described in Table 1 with materials, analytes, and detection methods such as amperometry, chronoamperometry, differential pulse voltammetry (DPV), square wave voltammetry (SW), electrochemical impedance spectroscopy (EIS), and cyclic voltammetry (CV), among others.

## 5. Challenges and Future Perspectives

As a result of the excellent properties of natural cellulose, such as being biocompatible, environmentally friendly, and versatile, cellulose-based electrochemical sensors offer an exciting alternative to state-of-the-art sensor technologies. These sensors have made striking progress in issues related to factors such as sensitivity and stability, thereby making them useful in various fields of possible applications like medical diagnostics, wearable technology, and environmental monitoring. However, several challenges persist in the full establishment of these sensors. Improvement in the quality of cellulose material is regarded as one of the main challenges. Indeed, the processes for the generation of high-quality nanocellulose are complicated and generally have low yields, hence raising difficulties for the elaboration of large-scale production routes. While such sensors demonstrate higher sensitivity in comparison with early types of sensors, further improvement in their selectivity and sensitivity to complex substances is still necessary for the satisfaction of a series of clinical and environmental demands. There is another issue: stability over time, because fouling, degradation, and environmental stress may impact sensor performance. Another problem is how to scale up production to make these sensors commercially viable while keeping costs low and performance consistent.

Some exciting possibilities soon will, therefore, be focused on the development of these sensors. Many other advanced nanomaterials, such as metal nanoparticles, will also be included in cellulose and may afford improved selectivity, stability, and sensitivity of the sensors toward a wide range of substances. Again, the sensors will be tailored for specific uses like medical diagnosis, environmental monitoring, and biosensing, keeping in view the requirements of the industry. The integration of such sensors with the Internet of Things (IoT) and artificial intelligence (AI) might enable the making of smart healthcare devices and real-time environmental monitoring systems. Furthermore, advances in manufacturing techniques are foreseen to allow for the optimization of such sensors regarding efficiency, scalability, and cost, thus enabling modern opportunities for their wide exploitation. Due to the eco-friendly nature of cellulose sensors and their potential for further improvement, they are poised to become at the forefront of sustainable, low-cost, high-performance sensing technologies. These sensors will further be able to revolutionize the sensing arena in several industries once duly developed.

## 6. Conclusions

In conclusion, due to their biocompatibility, environmental sustainability, and versatility, cellulose-based electrochemical sensors may provide a far broader range of potential applications than those of the predominant sensor types known today. These sensors have registered substantial improvements in the qualities of sensitivity, stability, and functionality, which have enabled them to be applied in medical diagnostics, environmental monitoring, and the wearable technology sectors. Nevertheless, optimization of cellulose materials is still needed, while there is a need to further improve selectivity and sensitivity for complicated analytes, along with long-term stability regarding real-life conditions. In addition, scalability is one of the important factors that will enable the commercialization of these sensors in widespread applications. However, the future of cellulose-based electrochemical sensors looks bright, with further advances in nanomaterial integration, sensor design, and synthesis techniques. The further development of such sensors, mainly through the incorporation of the Internet of Things (IoT) and artificial intelligence (AI), could provide cost-effective smart health devices and real-time environmental monitoring systems. Further research and innovation could position cellulose-based sensors at the forefront of sensing technologies’ future, offering an ecological and effective solution for a wide variety of applications.

## Figures and Tables

**Table 1:** List of cellulose-based electrochemical (Bio) sensors for the detection of varied analytes.

Materials	Analytes	Method of Detection	Reference
Microbial nanocellulose (MNC)/screen-printed carbon electrodes (SPCEs)	Pb^2+^, Cd^2+^, uric acid, and 17β-estradiol in artificial sweat	DPV	[154]
Reduced graphene oxide (NH_2_-rGO)/2,2,6,6-tetramethylpiperidin-1-yl) oxyl nanocrystalline cellulose (TEMPO-NCC)/screen-printed carbon electrode	Mycobacterium tuberculosis	DPV, EIS, CV	[155]
Silylated graphene oxide-grafted chemically modified nanocellulose (Si-GO-g-CMNC)/ZnO incorporated in CMNC/glassy carbon electrode	Cholesterol	CV, DPV	[156]
NCC/CdS quantum dots (QDs)/cetyltriammonium bromide (CTAB)/3-mercaptopropionic acid (3-MPA)/tyrosinase enzyme (Tyr)	Phenol	DPV	[157]
TEMPO-oxidized CNCs/glucose oxidase (GOx)/screen-printed electrode	Glucose	CV, chronoamperometry	[158]
Graphene paper/rGO/graphene oxide-coated nanocellulose/glucose oxidase (GOx)/RNA aptamer/chitosan	*E. coli* O157:H7, glucose	EIS, CV	[159]
Polypyrrole–cellulose nanocrystal/GOx	Glucose	CV, DPV	[160]
Graphene-perylene-3,4,9,10-tetracarboxylic acid nanocomposites (GR-PTCA)/nanocellulose–Au NP composites (NC-Au)/the alkaline phosphatase (ALP)	Avian leukosis virus subgroup J (ALV-J)	CV, DPV	[161]
Cellulose nanowhiskers (CNWs)/rGO/polyamide 6 (PA6) electrospun nanofibers	Hg^2+^	CV	[162]
Cellulose/pencil graphite electrode	Atrazine (ATZ)	SWV, CV	[163]
Nanocellulose/xanthine oxidase (XO)/glassy carbon electrode	Xanthine (XA)	DPV, EIS	[164]
CNCs/polyaniline (PANI)/glucose	Humidity, pH, organic solvents	Stimuli-responsive CNC/PANI composite films, electrochromic device	[165]
TEMPO-oxidized cellulose nanofibers-polyethyleneimine hybrids/single-walled carbon nanotubes (SWCNTs)	Tetracycline	CV, DPV	[166]
Thioctic acid (TA)/dopamine-grafted cellulose nanofibers (DCNF)/Li+	Strain, conductivity	Electrical conductivity measurement	[167]
CNCs/polydopamine (PDA)/AuNPs/polyvinyl alcohol/lactate oxidase (LOx)	Lactate in sweat	Lactate sweat sensor	[168]
Cellulose/KOH	Hydroquinone, catechol	CV, amperometry	[169]
Cellulose/polystyrene sulfonate sodium (PSS)	Ion concentration in sweat, pH	Cellulose-based conductive membrane	[170]
Cellulose hydrogel/AgNPs/ferrocyanide redox probe	Sulfamethoxazole	SWV	[171]
Cellulose nanofibers from wastepaper/1-(2-hydroxy-1-naphthylazo)-2-naphthol-4-sulfonic acid (HNNSA)	Co^2+^	Cobalt nanosensor	[172]
Cellulose/carbon nanotubes/glucose oxidase/Pt/Ag/AgCl	Glucose	Continuous glucose sensor	[173]

## Data Availability

Not applicable.
